# Born to Eat Wild: An Integrated Conservation Approach to Secure Wild Food Plants for Food Security and Nutrition

**DOI:** 10.3390/plants9101299

**Published:** 2020-10-01

**Authors:** Teresa Borelli, Danny Hunter, Bronwen Powell, Tiziana Ulian, Efisio Mattana, Céline Termote, Lukas Pawera, Daniela Beltrame, Daniela Penafiel, Ayfer Tan, Mary Taylor, Johannes Engels

**Affiliations:** 1 Alliance of Bioversity International and CIAT, via dei Tre Denari 472/a, 00054 Rome, Italy; D.Hunter@cgiar.org (D.H.); C.Termote@cgiar.org (C.T.); j.engels@cgiar.org (J.E.); 2 Center for International Forestry Research, Penn State University, State College, PA 16802, USA; bxp15@psu.edu; 3 Royal Botanic Gardens Kew, Wakehurst, Ardingly, West Sussex RH17 6TN, UK; t.ulian@kew.org (T.U.); E.Mattana@kew.org (E.M.); 4 Faculty of Tropical AgriSciences, Czech University of Life Sciences Prague, Kamýcká 129, 16500 Praha-Suchdol, Czech Republic; paweralukas@gmail.com; 5 The Indigenous Partnership for Agrobiodiversity and Food Sovereignty, c/o Alliance of Bioversity International and CIAT, Via dei Tre Denari 472/a, 00054 Rome, Italy; 6 Biodiversity for Food and Nutrition Project, Ministry of the Environment, Brasília-DF 70068-900, Brazil; dani.moura.oliveira@gmail.com; 7 Escuela Superior Politécnica del Litoral, Centro de Investigaciones Rurales–FCSH, Campus Gustavo Galindo-km. 30.5 vía Perimetral, Guayaquil 090112, Ecuador; ddpenafi@espol.edu.ec; 8 Faculty of Medicine, Universidad de Especialidades Espíritu Santo, Samborondon 091650, Ecuador; 9 Aegean Agricultural Research Institute, Menemen, Izmir P.O. Box 9 35661, Turkey; ayfer_tan@yahoo.com; 10 Environmental Studies, University of the Sunshine Coast, Maroochydore, QLD 4556, Australia; maryt@oxalis.plus.com

**Keywords:** wild food plants, food security, nutrition data, multi-sectoral collaboration, policy, conservation

## Abstract

Overlooked in national reports and in conservation programs, wild food plants (WFPs) have been a vital component of food and nutrition security for centuries. Recently, several countries have reported on the widespread and regular consumption of WFPs, particularly by rural and indigenous communities but also in urban contexts. They are reported as critical for livelihood resilience and for providing essential micronutrients to people enduring food shortages or other emergency situations. However, threats derived from changes in land use and climate, overexploitation and urbanization are reducing the availability of these biological resources in the wild and contributing to the loss of traditional knowledge associated with their use. Meanwhile, few policy measures are in place explicitly targeting their conservation and sustainable use. This can be partially attributed to a lack of scientific evidence and awareness among policymakers and relevant stakeholders of the untapped potential of WFPs, accompanied by market and non-market barriers limiting their use. This paper reviews recent efforts being undertaken in several countries to build evidence of the importance of WFPs, while providing examples of cross-sectoral cooperation and multi-stakeholder approaches that are contributing to advance their conservation and sustainable use. An integrated conservation approach is proposed contributing to secure their availability for future generations.

## 1. Introduction

The practice of consuming wild food plants (WFPs) is as old as human prehistory. Early humans obtained their food by hunting, fishing and gathering these plants, or parts of plants (e.g., stems, roots, flowers, fruits, leaves, buds, and seeds), that were safe for human consumption. It was not until 10,000 years BC that people started settling into more permanent homesteads and domesticating plant species (mostly carbohydrate-rich staples) while maintaining some hunter-gatherer activities and collecting WFPs from the wild [1,2]. This still holds true for some traditional horticultural societies today (e.g., the Machiguenga in South America) [3]. All of the plants we now call domestic crops were once WFPs, altered by human manipulation to achieve domestication by selecting more favorable plant traits. With plant domestication and farming came also the development of weeds; that is, unwanted plant species in cultivated fields, and many of the WFPs consumed today include relatives of today’s crops.

Today, the term “wild” is mostly taken to indicate species that grow spontaneously in self-sustaining populations outside cultivated areas, in field margins, forests, woodland, grassland, and wetlands (e.g., paddy fields), independently of human activity [4]. However, the distinction between “wild” and “cultivated” or “domesticated” is not so clear-cut and many WFPs fall somewhere in between these extremes depending on the degree of human intervention and management. For example, they can grow spontaneously in areas that are or have been themselves cultivated [4,5], or, as in the case of the “quelites” greens in Mesoamerica (e.g., the genus *Amaranthus*, *Chenopodium*, *Porophyllum*, *Portulaca*, *Crotalaria,* and *Anoda*), they have become the focus of systematic in situ management practices such as “selective harvesting” and “let standing”, with important repercussions on plant communities [6]. Another known management practice is that of “encouraging growing” recorded by Cruz-Garcia [7] in the Peruvian Amazon along the deforestation border. Surveys revealed that, out of 30 wild food plant species identified, 20 are actively managed by local farmers and that most are transplanted from the forest to their agricultural fields for easy access. From these, 57% of the species are classified as weeds, yet are perceived by farmers to play a role in food security, particularly with increasing deforestation and reduced availability of food plants [7].

In this review paper, the term “wild food plants” is extended to all those food plants (herbs and spices included) that are also semi-domesticated, in addition to economically important non-timber forest food products, such as açaí berries and Brazil nuts [8]. As they are often wild relatives of domesticated species, WFPs have potential for domestication and can provide a pool of genetic resources for hybridization and selective breeding [9].

## 2. The Importance of Wild Food Plants Today

WFPs continue to play a vital role in the subsistence of many human populations particularly when the availability of food crops is scarce, when household budgets are insufficient to buy enough food or when access to markets is challenging [5,8,10,11,12,13,14,15,16]. Wild foods are also integral to traditional food systems and have nutritional and cultural value for many indigenous peoples [4,5,17,18]. Deeply connected to their land, indigenous peoples, who represent 5% of the global population [19], are often the sole custodians of rich and diverse knowledge relating to plant uses and traditional food systems and to local food biodiversity existing within the ecosystems they inhabit [18]. Traditional communities also have better ecological knowledge about local environments and their customary users, making monitoring and regulating of natural resources easier [20].

Although the caloric contribution of WFPs to people’s diets is generally low compared to staple foods [21], these species contribute to diet diversification in many geographical settings where otherwise monotonous diets may prevail [22,23,24,25,26]. Wild foods (both plants and non) provided between 1% and 19% of the iron consumed, between 5% and 45% of the calcium and between 0% and 31% of the vitamin A equivalents (RAE) in the diets of women and children in studies from Benin, Tanzania, and the Philippines [21]. These neglected biological resources have, in fact, been shown to contain equally, if not higher amounts, of nutrients than more widely available commercial crops [5,27,28,29], and, if properly assessed and managed, could be introduced in national food and nutrition security and sovereignty strategies that focus on nutrient adequacy rather than quantity of staples, while being culturally acceptable.

WFPs could also be central to efforts directed at empowering local market actors as well as reducing the distance between consumers and producers and the overreliance on globalized value chains. Although, recent research by Kinnunen et al. [30] highlights the unfeasibility of localizing production for important global staples such as rice, maize and temperate cereals, there is increasing evidence that the local trade of minor crops, traditional varieties, and WFPs has potential to empower communities and increase livelihoods in rural areas, particularly of women and youth [31,32]. Meanwhile, the COVID-19 crisis has revealed the vulnerability of our global food systems to disease-related disruptions and shocks [33,34,35]. For example, the imposed travel restrictions on people and goods as a result of the lockdowns are causing logistical bottlenecks in food supply chains [36]. Given the national and international trade restrictions, long supply chains are struggling to cope with the rise in food demand for non-perishable food supplies [37], while short supply chains are suffering due to the closing of informal and local open-air markets [38], where the majority of the world’s population still obtains fruits, horticultural, and other perishable products [37,39]. At the same time, the pandemic has opened up opportunities for a new food system paradigm that supports local self-sufficiency and domestic agricultural production and sees home and community gardens, traditional agroecosystems, and farmers’ markets as essential services [38,40]. With food shortages affecting specialized, high value horticultural crops [41], people are turning to traditional vegetables and WFPs as a sustainable source of food, vitamins and nutrients [42], not to mention for herbal ingredients, traditional medicine formulations or new biopharmaceuticals [38,43,44].

This paper builds mainly on the authors’ own efforts being undertaken in several countries to provide evidence of the role of WFPs in supporting nutrition and livelihood security. This paper also provides examples of cross-sectoral cooperation and multi-stakeholder approaches that are contributing to the better conservation and use of WFPs, including by fostering linkages between in and ex situ conservation. In the case of WFPs, “use” includes the various practices and activities involved in (i) domesticating wild species; (ii) the management of wild species and their habitats in and around production systems to promote the delivery of ecosystem services; and (iii) the introduction of wild species into production and consumption systems, for example by creating demand for the species, and regulating their harvesting in the wild. Lastly, details will be provided of a proposed integrated conservation approach that focuses on local interventions based on traditional food systems.

### 2.1. Diversity (Geographical Use) and Contribution to Diets

The use of WFPs in many countries is confirmed by national contributions to the recent “State of the World’s Biodiversity for Food and Agriculture”—”SOWBFA”—of the Food and Agriculture Organization of the United Nations [45]. Of 91 countries reporting information for compiling the report, 69 nations reported a total of 1955 wild plant species that contribute to food security and nutrition in their respective countries, as well as making diets healthier and more diverse. However, as the examples provided by the authors and recently published papers [46] demonstrate, the number is probably much higher and these species remain largely unreported in national statistics, as does the actual contribution of these biological resources to national economies in many parts of the world [47]. Table S1 in the Supplementary materials lists the wide range of plant families that encompass the edible wild and semi-cultivated plant species researched by the authors and mentioned in the text as contributing to food and nutrition security. The list, as the review carried out by the authors, is by no means exhaustive and could undoubtedly include more.

#### 2.1.1. Africa

As part of the MGU Useful Plants Project (UPP) managed by the Royal Botanic Gardens, Kew, UK, institutional partners working alongside local communities in Botswana, Kenya, Mali, South Africa, and Mexico identified 615 species used for food across the five countries [48]. Information on seed conservation, propagation and traditional uses has been published for 48 of them and is now available on the internet [49]. In Africa, the species included the baobab (*Adansonia digitata)*, the mongongo tree (*Schinziophyton rautanenii*), and the morama bean (*Tylosema esculentum*) [49]. Research undertaken by Bioversity International in the early 1990s has documented 210 African leafy vegetables in Kenya alone [50]. These are wild or semi-domesticated species that are grown mostly for household consumption or traded informally, but which have seen a revival particularly in urban and peri-urban areas [51]. In Western Kenya, between 23 and 42 African leafy vegetables continue to be consumed by local communities depending on the district. Eleven species, including amaranth (*Amaranthus* spp.), spider plant (*Cleome gynandra*), and African nightshades (*Solanum* spp.) were selected for further research as part of the African Leafy Vegetables program from 1996 to 2004 (Bioversity International and EIARD, 2013; Gotor and Irungu, 2010) [51,52] as well as for the Biodiversity for Food and Nutrition Project [28,53]. In addition to filling the nutrient gap, a cost of diet study carried out in Eastern Baringo, Kenya, has shown that wild plant species, especially vegetables, are able to significantly reduce (by 30–70%) the cost of a nutritious meal for women and children aged 6 to 23 months in hypothetically-modeled lowest cost nutritious diets [54].

#### 2.1.2. South America

In Ecuador, one of the top seven mega diverse countries in the world, wild edible fruits and plants collected from a diverse range of habitats play a fundamental role in traditional diets, particularly for the indigenous communities living in forest areas. Studies in the country by Penafiel et al. [55,56], documenting local knowledge on the use of WFPs among the Andean indigenous communities of Guasaganda (Cotopaxi) and the Andean Kichwa mothers of Arosemena Tola (Napo), recorded the culinary use of 49 and 10 WPFs, respectively. Brazil also contains vast amounts of wild food plant diversity [57]. Some of this diversity is of national and regional relevance, e.g., Brazil nut (*Bertholletia excelsa*) and açaí (*Euterpe oleracea*), but most is of local value and its potential nutritional and economic value remains unexplored and unexploited [58]. The “Plants for the Future (PPF) Initiative”, a prioritization exercise undertaken by the Ministry of the Environment that set out to explore the wealth of Brazil’s plant biodiversity, has identified a considerable number of wild species of nutritional value and market potential. Across the country’s five eco-regions, out of 78 native undervalued edible plant species, 49 are found exclusively in the wild (mostly fruits and nuts) [28]. Mostly found in forest areas, the species are managed by family farmers or harvested from the wild by local communities using traditional practices. The link between local communities and nature is such that the Brazilian ministries of Agriculture, Environment, and Social Development have coined the term “sociobiodiversity” to describe these traditionally managed biodiversity-derived goods that are sold in local markets, provide incomes and improve the livelihoods of traditional communities, while protecting biodiversity and the environment.

#### 2.1.3. The Mediterranean

In the Mediterranean, WFPs are still common in traditional cuisine and are widely consumed locally [59,60]. In their compendium of gathered Mediterranean food plants, Rivera et al. [61] identified approximately 2300 different WFPs and fungi taxa in this region alone, of which 1000 are strictly used locally. As part of the Biodiversity for Food and Nutrition (BFN) project, Turkey prioritized 42 wild edible plants for further research [28] out of hundreds of known species [59,62,63], while across Morocco, Nassif and Tanji [64] compiled a list of 246 wild plant species used as food. While many WFPs are only used occasionally or in small regional areas, some are central to Moroccan diets and culinary traditions. Aromatic herbs such as thyme (*Thymus* spp.), mint (*Mentha* spp.), and sage (*Salvia* spp.) are the most widely consumed wild plants; however, they contribute little to the diet in terms of energy (kcal) and nutrients because they are used as condiments [65]. Wild leafy vegetables, on the other hand, are a seasonally important component of Moroccan diets, particularly in rural Morocco where 86% of households reported consuming wild leafy vegetables (WLVs) on a regular basis [66]. Some of the most commonly consumed WLVs (many of which are also consumed in Turkey and other Mediterranean countries) include mallow (*Malva* spp.), purslane (*Portulaca oleracea)*, goosefoots (*Chenopodium* spp.), docks and sorrels (*Rumex* spp.), fennel (*Foeniculum* sp. cf *F. vulgare*), golden thistle (*Scolymus hispanicus),* and watercress (*Nasturtium officinale)*. They are commonly served as a cooked salad or side dish, eaten in moderate portion sizes (approximately 50 g per meal). Argan oil (*Sideroxylon spinosum*), capers (*Capparis spinosa* and *C. decidua*), acorns (*Quercus* spp.), and the fruits of the strawberry tree (*Arbutus unedo)* as well as jujube (*Ziziphus jujube*), mulberry (*Morus* spp.), and blackberry (*Rubus* spp.) are other commonly consumed plant products in this region [64,65,67].

#### 2.1.4. Asia Pacific

The consumption of WFPs and food trees makes a significant contribution to human health in the Pacific region [68]. In “Food Plants of Papua New Guinea, A Compendium” [69], Bruce French produces a list of food plants, many of which are sourced from the wild, including root crops and staples, legumes, green leafy and other vegetables, nuts, fruits and what are categorized as “minor foods and flavorings”. For example, the kernel of wild *karuka* (*Pandanus brosimos*), endemic to Papua New Guinea (PNG), is eaten by about one-third of the rural population [70], particularly by communities living at high altitudes. When the fruit matures, villagers migrate to high altitudes to harvest the fruit and extract the nuts. Nuts have not been recorded in the main highland markets, but it is possible that they are sold in some high-altitude locations [71]. PNG and surrounding region are also one of the few places in the world where communities obtain the majority of their carbohydrate staple from a wild food plant: sago [72,73].

In Niue (Polynesia), the traditional processing of wild arrowroot (*Tacca* spp.) is still an ongoing practice. Starch processed from the root is a local delicacy used to make local puddings and breads [74]. Thaman [75] lists 60 WFPs used in Fiji, noting that these plants play an important role as emergency or famine foods when extreme climatic events disrupt cultivation. Among these are wild marine seaweeds such as sea grapes (*Caulerpa racemosa*), known as nama, and other edible seaweeds that are still widely consumed. “The Guide to the Common Edible and Medicinal Sea Plants of the Pacific Islands” provides an insight into marine WFPs and the benefits that can be gained from their use [76].

In the mosaic tropical landscape of West Sumatra, Indonesia, composed predominantly of rice fields, home gardens, cacao agroforestry, and forests, with the help of local communities, the Food, Agrobiodiversity and Diet (FAD) project has identified 85 WFPs [77]. In this region, WFPs are consumed less than in the past, and the FAD project aimed to raise knowledge and awareness of wild foods by organizing workshops, traditional food competitions, and sharing community materials such as illustrations, posters, and community guidebooks, on food plants for nutrition and health [78].

### 2.2. Income Generation

In many parts of the world, WFPs are not only harvested for subsistence [79,80,81]. Gathered in excess, they are sold in local markets to generate income, thereby contributing to the household economies of gatherers and collectors, usually women, or to bolster the incomes of migrants and unemployed moving from rural to urban areas [82]. For example, in the Chimanimani communities living in the Trans-Frontier Conservation Area in Mozambique, the fruits of *Uapaca kirkiana* and *Strychnos madagascariensis* are sold for a reasonable profit and represent an important source of income outside the maize harvest season (March to May) [83]. In their review paper, Hickey et al. [84] showed that 50% of almost 8000 households sampled in forested areas of 24 developing countries across Asia, Africa, and Latin America derived their income from wild food collection. The study also highlighted that the sale of plant foods contributed 2.3% to total household income across the study sites on three continents, the proportion increasing to 2.8% in Africa and Latin America, particularly in poorer households.

In parts of Turkey, where WFPs are central to traditional cooking, wild edibles are sold unprocessed in local markets and processed (e.g., pickled, canned, or frozen) in district markets or supermarkets via wholesalers and middlemen [85]. In 2012, in the Pacific Island States of Fiji, Samoa, and Tonga the yearly production and revenue from the harvesting and sales of the seaweed *Caulerpa racemosa* was valued at USD 266,492 [86]. However, the true extent of this revenue is not always available. For example, a recent European assessment established the value of collected non-wood forest products, mainly food plants, at € 19.5 billion with value per hectare rising to € 77.8, and ten times above the official European estimates [87]. Many markets for WFPs are informal, and market players may hold back information because of illicit harvesting in local conservation areas [82]. Data about geographic and temporal distribution, production cost, quantity harvested, and price is also often limited. Increased profits can often lead to overexploitation of WFPs and negative outcomes for the entire community [20]. To avert this possibility, participatory research is key to establish sustainable management guidelines and harvest rates, and to monitor the ecological impacts of increased use [83].

### 2.3. Threats to WFPs

**Figure 1:**
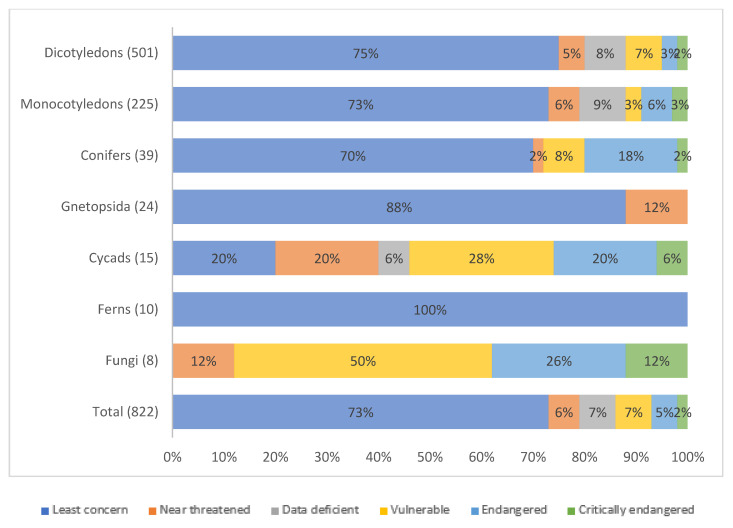
Number of WFPs and fungi on the IUCN Red List of Threatened Species classified by class and risk category Source: IUCN Red List 2017. Adapted from FAO [45].

Despite the realization of the potential use of WFPs in food security and poverty reduction strategies, the SOWBFA, along with other recent global reports [46], warn us that this precious diversity is fast disappearing, particularly in forest habitats [88,89]. Land use changes (e.g., conversion to agriculture, change in agricultural practices and infrastructure development), habitat destruction (resulting from timber harvesting, fuelwood collection, grazing, and forest fires) and overharvesting collectively account for 62% of the threats reported to WFPs in SOWBFA, which mostly grow beyond the limit of protected areas [45,90,91]. The SOWBFA used the Sampled Red List Index for Plants of the International Union for Conservation on Nature (IUCN) [92] to determine that, of a total 822 WFP species considered across 7 different classes, 73% are currently at low risk of extinction (Figure 1), with some classes highly threatened in the wild (e.g., WFPs that are derived from conifers and cycads). However, the IUCN Red List Index for Plants includes global conservation assessments for only one third (31%) of known WFPs. Local assessments for many WFPs that are currently excluded from the IUCN assessment paint a very different story indicating the need to consider community perceptions when ascribing risk class (Table 1). Furthermore, an assessment of the comprehensiveness of conservation of 1587 WFP taxa (including cereals, fruit, and nuts), carried out by the International Center for Tropical Agriculture (CIAT) as part of a larger study to identify conservation gaps for useful plants, shows that only 3.3% of WFPs are sufficiently conserved ex situ, i.e., in gene banks or in other living plant repositories, while 89.1% require urgent off-site conservation measures given the impending threats to their existence [93]. Their continued use in diets, when accompanied by careful sustainable management by the communities consuming them, and protection of WFP habitats, on the other hand, seems to have ensured their momentary conservation in situ, in the natural habitats in which they grow. Of the WFP taxa analyzed 42.1% are sufficiently conserved, 46.7% deserve medium priority and 11.1% require stepping up conservation measures [93]. Nonetheless, Khoury et al. [93] caution against the overreliance on protected areas for the long-term conservation of these species. Rapidly warming temperatures and habitat destruction can alter the species’ geographic distribution, driving them across the artificially designated boundaries of many protected areas in pursuit of favourable growing conditions [94].

Given that many WFPs grow in agricultural systems (as weeds, in hedge rows, as wild trees in agroforestry systems, and in small forest patches [5,21]), agricultural change, including intensification, more pesticide use and removal of trees can threaten the existence of these biological resources [12,14,95]. Food production systems that pollute the environment by using large quantities of fertilizers, pesticides, and herbicides, are also major causes of biodiversity loss [45,88,96]. Applying chemical herbicides in rice fields or agroforestry plots, for example, leads to the reduced availability of WFPs in West Sumatra, Indonesia [77]. WFPs that survive aerial spraying with herbicides are contaminated by these harmful substances, making them unfit for human consumption, while pesticides wipe out many of the pollinators needed for plant reproduction.

Overharvesting can also be an important pressure on non-timber forest products, including wild foods [97]. This is the case for Morocco and Turkey. Morocco is the twelfth global exporter of medicinal and aromatic plants, a trade that places extensive harvesting pressure on many of the species traditionally used as herbs [98]. A rapid vulnerability assessment carried out by Lamrani-Alaoui and Hassikou identified six species that grow across wide areas of Morocco’s government owned forests (*Thymus satureioides, Lavandula dentata, Origanum compactum, Origanum elongatum, Salvia rosmarinus* and *Fraxinus dimorpha*) as needing urgent conservation, restoration, and sustainable management measures [98].

Exacerbating these problems in the different geographies are the uncertain effects of climate change, which in many countries is expected to lead to increased variability in seasonality, temperatures and precipitations and increased incidence of hurricanes and wildfires [89]. Climate change is also predicted to severely impact cultivated plants, affecting crop production in specific geographic locations [115], stripping nutrients from staple crops [46,116] and making WFPs all the more important for food and nutrition security. Although generally highly adaptable and often more drought tolerant than cultivated crops, WFPs, as many useful plants, are also not fully resistant to climate change [116]. In the past, many WFPs survived major climatic fluctuations, but thematic studies on the implications of future climate change suggest important impacts on the ability of wild species to survive. This includes WFPs, particularly in tropical regions where economies are already fragile and capacity may be inadequate to protect these species effectively [94,116]. One key impact that could threaten WFP use is the likely shifts in both WFP geographic ranges and phenological changes in ripening times. This could create mismatches with traditional knowledge and practices of the communities that traditionally harvest them [117].

At present, there are very few formal systematic efforts that support and regulate the conservation and sustainable use of WFPs [118]. A sample survey of some of the most recent National Biodiversity Strategies and Action Plans (NBSAPs) submitted to the CBD as part of the reporting requirements of member states (e.g., Chile, Morocco, and Portugal), shows that rarely do these strategies refer specifically to WFPs or, if they do, are very vague in terms of the measures needed to protect them. Actions are mostly limited to ex situ conservation measures [46], while no concrete activities are put forward to support their conservation via sustainable use [45]. Furthermore, appropriate and effective governance mechanisms are seldom in place to safeguard the rights of indigenous people and local communities to sustainably manage and benefit from the use of WFPs (and prevent their over-exploitation by others) [119].

The use of wild species, however, is explicitly recognized as useful for improving food and nutrition security in several international agreements, strategies and action plans: in the 2030 Agenda for Sustainable Development (SDG2, Target 2.5), the International Treaty on Plant Genetic Resources for Food and Agriculture (International Treaty), the Second Global Plan of Action for Plant Genetic Resources for Food and Agriculture (Second GPA), and in the Global Strategy for Plant Conservation of the Convention on Biological Diversity (CBD). The CBD, the main international agreement aimed at conserving biological diversity, accords explicit recognition to sustainable use for the long-term conservation of ecosystems, species and genes, which must continue to be used, but “in a way and at a rate that does not lead to the long-term decline of biological diversity” [120]. Intrinsic in the term “sustainable use” is that it generates benefits (e.g., nutritional, cultural, and financial) for the custodians and users of these wild species. These benefits encourage people to continue conserving these biological resources and the habitats in which they grow or live. However, the real challenge is to ensure this sustainability is maintained given the rising demands on global resources imposed by population growth and economic development, combined with the uncertain effects of climate change mentioned above.

## 3. Barriers to the Greater Use of WFPs

The disregard of WFPs for food security and nutrition can be partly attributed to a lack of evidence and awareness among policymakers and other stakeholders of the importance of wild foods to diets, livelihoods, and food security, coupled with a number of market and non-market barriers limiting their untapped potential.

Underpinning the lack of recognition for WFPs is also limited or short-term research and extension funding to support the exploration of non-conventional, traditional and indigenous food resources. Many of these barriers were summarized by Heywood [4] and are still very much valid today:

- lack of information about the extent of their use and importance in rural economies;
- lack of information, especially statistics, concerning the economic value of WFPs;
- lack of reliable methods for measuring their contribution to farm households and the rural economy;
- lack of information on the sustainability of current harvest levels;
- poorly developed infrastructure and markets for WFPs, with the exception of small number of products (e.g., Açaí berries);
- unevenness of supply;
- lack of quality standards;
- general lack of storage and processing technology;
- availability of substitutes;
- policies and research mostly favoring commodity crops and commercial agriculture.

Like other neglected and underutilized species, additional barriers to the promotion of WFPs in food production and consumption patterns include: limited and fragmented data of the nutritional importance of these species; fragmented data on the quality and nutritional impacts of WFPs on household nutrition [121]; and knowledge gaps on the species’ biology and ecology to develop domestication and management strategies [45,46].

Unfavorable and disabling national policies, coupled with the many stakeholders and interests involved, represent an additional obstacle to greater recognition for WFPs. The main policy barriers were identified and summarized by the Strategic Framework for Underutilized Plant Species [122], of which WFPs are part of. These are provided in Table 2 below.

Further contributing to the demise of WFPs, is the low recognition of value and perception of these foods as being “women’s food” [66] “food for the poor” or “famine foods” to be harvested only when staple crops fail, as well as lack of institutional capacity to mainstream this diversity into national production and consumption patterns [28]. On the other hand, in some regions, such as West Sumatra, communities perceive WFPs positively, but the main barrier to their greater use is their reduced availability caused by land degradation and agriculture intensification [77]. In many places, traditional wild leafy vegetables are disappearing from local diets due to changing dietary patterns and preferences driven by globalization and increasing market integration [123]. Wild leafy vegetables (WLV) and wild food plants in general are undervalued and seen as “un-modern” in Morocco, Turkey [28,66], and many other parts of the world. This lack of value places the role of WFPs in the diet at risk, although it may ease pressures on overharvesting. In Brazil and Kenya, changing dietary patterns and lifestyles has reduced the diversity and availability of wild fruit and vegetables in market settings, which focus instead on a limited number of exotic crops [124]. This has led to people consuming sub-optimal diets that are increasingly unhealthy, unsustainable, and inequitable for many populations [125].

### 3.1. Contribution to Nutrition and Diets

The contribution of wild food biodiversity to diets and nutrition is simultaneously limited by a severe lack of food composition data for many neglected and underutilized cultivated and wild foods [126] as well as by a lack of accurate botanical identification for many foods recorded in dietary records or food composition tables [45,127,128]. Nutrient composition data indicates the presence and quantity of nutrients (e.g., energy, proteins, minerals, and vitamins) as well as the compounds that can impact the bioavailability of nutrients within a food. These data are combined with dietary records of the foods consumed to assess whether individuals or groups are meeting their dietary requirements [129]. Nutrient composition data do not exist for many WFPs, and when they do there may be high variation in nutrient composition for a given species across space and time [130]. The few WFPs that have nutrient composition data and that are included in local food composition tables are often identified by local names. This hinders the use of these data to fill nutrition gaps in other locations where the same species might be present and used but is identified by a different local name. Many data sets lump all wild foods into a single food group (e.g., wild greens). For example, in analyzing data on wild harvests in 24 developing countries across Africa, Asia, and Latin America, Hickey et al. [84] found that only a small percentage (0.9%) of the collected mushrooms were identified by species, the rest was reported nonspecifically as ‘‘mushrooms”.

In some cases, when WFPs are lost from the diet they may be replaced by similar cultivated species, but in other cases they are not. Anecdotal evidence from Morocco suggests that when people stop or reduce the consumption of WLVs in their diet, these are not replaced with cultivated alternatives, leading to a reduced consumption of any leafy vegetable and fruit and vegetables in general. This is particularly worrying given global recommendations [131] to consume at least 5 servings of fresh fruit and vegetables (including berries, green leafy and cruciferous vegetables, and legumes) per day as a protective measure against cardiovascular diseases and type II diabetes [132,133,134,135,136].

Practical challenges also exist in measuring wild food consumption and contribution to the diet relative to other foods [5,137]. Although in recent years, several investigations have tried to assess the role of wild food biodiversity and the contribution of forests and agroforestry systems to human dietary intakes [13,14], the real dietary contribution of wild food plants, berries, fruit, nuts, and mushrooms harvested within and around people’s homesteads and in forested areas remains poorly understood. Geographical variations exist regarding the proportion of WFPs consumed. While in in the global North WFPs mostly have cultural and recreational value [138], in some low-income countries they significantly enrich people’s diets [119]. Rowland et al. [13] found that the collection of forest foods represents a regular livelihood strategy for many households and that forest dependent communities living in specific sites in Brazil, Cameroon, and Ethiopia derive as much as 80–96% of wild fruits and vegetables from the forest. In some areas, the nutritional contribution of fruits and vegetables is such to cover 50% and above the minimum dietary recommendation for these food groups [13]. Differences in consumption might also vary by ethnicity. For example, in documenting the traditional food systems of Western Sumatra, Pawera et al. [77] found that different ethnic communities living in the same environment have different knowledge and uses for the same WFPs. Seasonal fluctuations in WFP occurrence and therefore consumption by local communities might also not be adequately captured with a single survey [139]. Other challenges include cultural and language barriers and perceived power imbalances during questionnaire administration that can alter the surveys’ accuracy and reliability [137]. There is a huge body of research that only lists the edible species known to community members but neglects to quantify the use of WFPs in local recipes nor is their use standardized in nutrition surveys [121].

### 3.2. Gathering Grounds, Collection Practices and Use

An additional knowledge gap is represented by the lack of information on traditional gathering grounds and the sustainability of collection practices. In the SOWBFA, the ecosystem origin reported for WFPs is either from forests (>25%) or unknown (>45%) [45]. An often-overlooked practice is urban and peri-urban foraging for WFPs. In their cross-continental study of urban foraging spanning India, South Africa, Sweden, and the US, Shackleton et al. [140] found that urban foraging is a widespread custom that is practiced independently of wealth and social status and is driven by different motivations varying in time and place. Wooded areas on public land, local lake beds, and other urban habitats harbor nutritionally rich greens and fruits. Even spontaneous vegetation growing in alleyways was reportedly used by Indian residents for food and culinary use [140]. Aside from direct consumption and small-scale trade, other benefits include “improved physical and psychological health, sense of place, increased ecological knowledge, stronger connections with nature, food, income or cash saving, and a source of pride” [140]. The important cultural ecosystem services offered by these plants are apparent in a study of WFP gathering and consumption trends across Spain [141]. The authors observe that WFPs continue to be used in areas with deep-rooted culinary traditions and in some instances have become gourmet ingredients for chefs. Schulp et al. [142] also suggest that the cultural benefits of wild foods in the European Union might exceed their income and food benefits and observe that wild mushroom and food plant collecting are generally highest in Southern European countries where gastronomic identity is strongest.

## 4. An Integrated Approach for Conserving and Sustainably Using WFPs

**Figure 2:**
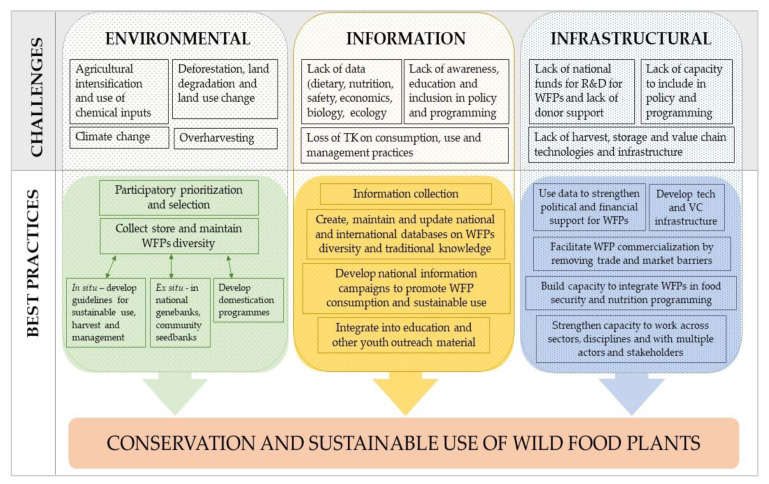
Proposed best practices for the long-term co-creation of conservation and sustainable use of WFPs help overcome many of the challenges identified.

With the gradual disappearance of WFPs from nature and diets, the question is how to effectively promote their sustainable use and simultaneously conserve them for food security and nutrition. Because they exist on a continuum of human management, from truly wild to semi-domesticated [7], and because the germplasm and other plant material (e.g., tissue, embryos etc.) of some species may not be suitable for ex situ conservation [143], both in situ and ex situ conservation should be combined for optimal results [144,145,146] (Figure 2). In situ conservation strategies can complement ex situ conservation and allow WFPs to continue to evolve adaptive traits in their natural environments while benefiting those who need them most, particularly in areas where high diversity, rural poverty and malnutrition coexist.

Above, we have identified an array of threats to WFPs including: land use changes, deforestation and degradation; agricultural change, intensification and chemical input use; overharvest or unsustainable harvesting; loss of traditional management practices that communities used to promote the production of wild food plants (for example, pruning and burning); and climate change. We also identified a range of barriers that are contributing to the loss of use and value for WFPs, such as, lack of information (diet, nutrition, safety economics, and ecological); lack of harvest, storage and value chain tech and infrastructure; and lack of awareness, education and inclusion in policy and programming. In the subsequent sections of this paper we propose a set of best practice actions that can be taken to support sustainable use and conservation of WFPs. This set of actions laid out in Figure 2 will act to overcome or mitigate against many of the threats and barriers identified.

The proposed set of best practice actions includes: the collection of information (identify the diversity of WFPs that are present in a given environment, information on nutrient composition and contribution to diet, economic importance, and ecological studies to determine sustainable offtake); (ii) prioritize the species with greatest potential to fill nutrition gaps, greatest need in terms of conservation, greatest cultural importance; (iii) protect species that are vulnerable through ex situ conservation; (iv) promote the use and management of WFPs in natural environments (in situ) (including sustainable management and collection guidelines where needed); (v) develop domestication programs where necessary and possible to avoid overexploitation in the wild; (vi) build local capacity to improve storage, processing, value chains, and markets (and all related technology and infrastructure); (vii) integrate WFP into programming and education and other youth outreach so as to raise awareness; (viii) develop and strengthen policies that support the conservation and sustainable use of WFPs; and (ix) and build donor commitment to funding efforts to support sustainable use and conservation of WFPs.

Each community and each WFP are unique, and will require a different set of actions, possibly occurring in a different order. Successful implementation of the set of best practice actions best suited to any given context will require working in a coordinated fashion across disciplines and sectors at the local, regional, and international level, and is largely dependent on the close and active participation of the national and local stakeholders. Due to the limits of time-bound projects (e.g., capacity, resources), it is rare for a single project or intervention to cover all elements or actions needed for a comprehensive and integrated approach. Below we present examples of best practice actions that we believe have successfully helped to further the conservation and sustainable use of WFPs.

### 4.1. Identify and Prioritize

**Figure 3:**
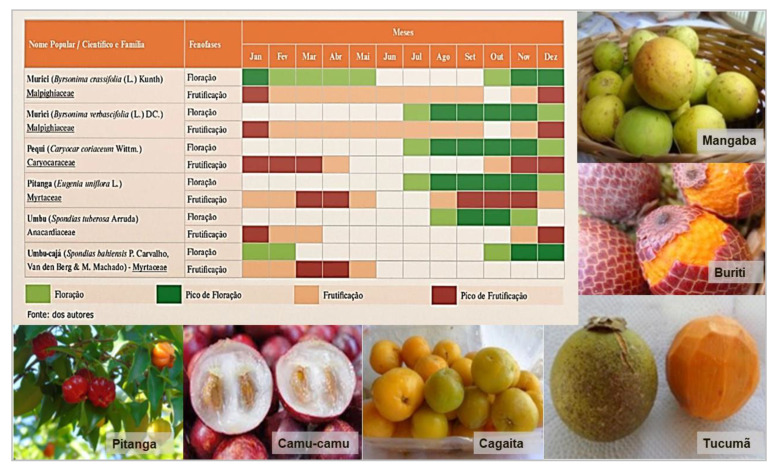
Research into the flowering and fruiting period of wild fruits and greens within a given geography can be used to develop an adaptable tool for informed decision making at both community and government level. Credit: BFN Brazil.

The identification of WFPs to include in conservation and sustainable use strategies will almost invariably require close collaboration with indigenous and local communities who are the main users and custodians of this diversity. As opposed to extractive methods, participatory research approaches that integrate traditional and scientific knowledge are the most appropriate to collect information on WFPs while maximizing benefits for the communities involved [147]. Prior to the intervention, the community should be aware and agree on every aspect of the research process so that the methods, the analysis and the purposes of the data collection are clearly understood [148]. Ethnobotanical surveys and free-listing exercises are the most commonly used methods to complement scientific ecogeographic assessments. In the majority of the studies discussed in this paper, focus group discussions conducted with knowledgeable key informants were able to help fill knowledge gaps in WFP availability and use. Useful tools for assessing the potential of WFPs to fill seasonal food insecurity gaps, and low dietary diversity characterized by low fruit and vegetable consumption, are seasonal calendars, such as the one shown in Figure 3 developed by BFN Brazil to investigate flowering and fruiting seasons for wild fruit species. Data can then be transformed into an accessible and understandable tool to assist communities and decision makers adopt healthier diets based on local biodiversity [149].

Market surveys are also useful to understand what WFPs might be available for consumption within a community. A notable example is represented by BFN Turkey, which undertook market surveys and key informant interviews with 2334 local wild plant gatherers, sellers and consumers of WFPs to capture the diversity of WFPs still being used across three ecogeographic regions [28,150]. Documenting the use of wild plants in this participatory way has several benefits that include: (i) facilitating knowledge transmission from elders to younger generations and between community members; (ii) stimulating local innovation without undermining cultural traditions and local governance mechanisms, and (iii) ensuring that the community can use this diversity to address its own questions, challenges and needs [147]. In Western Kenya, for example, biodiversity surveys and dietary health assessments were followed by a series of participatory workshops that enabled five communities to gain and share knowledge on available wild and cultivated biodiversity, discuss options on ways to use this diversity to improve malnutrition within their communities, rank and prioritize the most suitable species and develop their own community action plans (CAP) towards this end. In collaboration with the ministries of Agriculture and Health, training was then provided to assist with the integration of the chosen species—mostly African leafy vegetables and legumes—into sustainable production systems and diets. One year into CAP implementation, mean dietary diversity scores and the percentage of children meeting minimum dietary diversity had significantly increased in all the households in the sublocation, irrespective of participation in the scheme, indicating the adoption of these best practices by neighboring households. The dietary diversity scores of women from participating households had also significantly increased [151].

With all probability, surveys will reveal a long list of species that could be the focus of further research and promotion in food and nutrition strategies. Realistically, limited resources will often require a prioritization exercise that reduces the list to a manageable number. Since the intent is to ensure that WFPs are safeguarded and sustainably consumed, again community participation in the prioritization process is key, for example, to single out species that could be conserved in seed saving facilities, domesticated, or included in breeding programs, or to identify WFPs with the potential to contribute to nutrition, climate-change resilience and other aspects of community well-being. In the earlier example from Turkey, the BFN team developed an ad hoc sustainability index to reduce an initial sample size of 43 species, mostly WFPs, to three target species—foxtail lily (*Eremurus spectabilis*), golden thistle (*Scolymus hispanicus*), and einkorn wheat (*Triticum monococcum*)—which have since been the object of domestication research as well as post-harvest handling and value chain analysis [150].

### 4.2. The Nutritional Importance of WFPs and Associated Traditional Knowledge

Understanding the nutrient content and health properties of WFPs (e.g., compositional data) as well as their contribution to diets will also greatly assist in the prioritization process. Compositional data is key to national nutritional planning and for developing locally fitting dietary guidelines. Seldom, however, does nutrition information appear in national food composition tables and databases, and if data does exist it is either scattered across different sources in institutional databases, in academic journals and grey literature, or is outdated and/or incomplete making data compilation a daunting task [28,150]. An additional hurdle is the standardization of food composition values. Common component names are often expressed inaccurately (e.g., vitamin A: retinol activity equivalents vs. retinol equivalents) or differ in terms of units, denominators, significant figures, maximum decimal places, and conversion factors [152]. Of further importance, is the documentation and protection of traditional knowledge related to consumption and preparation of WFPs, available largely in the recollections of elderly users, i.e., rural, indigenous, and forest-dependent communities, including local farmers, and city migrants. Some information may be available in national floras, in herbaria and in ethnological studies of local human ethnical groups, but additional botanical, culinary, nutritional, cultural research is required to fill this knowledge gap. As explained in Section 4.1, to avoid issues of misuse and abuse of this information, it is important that respondents are always adequately informed about data use, that sources are acknowledged, and that the data is made available in public databases. Biological knowledge on individual species is also frequently lacking but particularly essential for both in situ and ex situ conservation.

**Figure 4:**
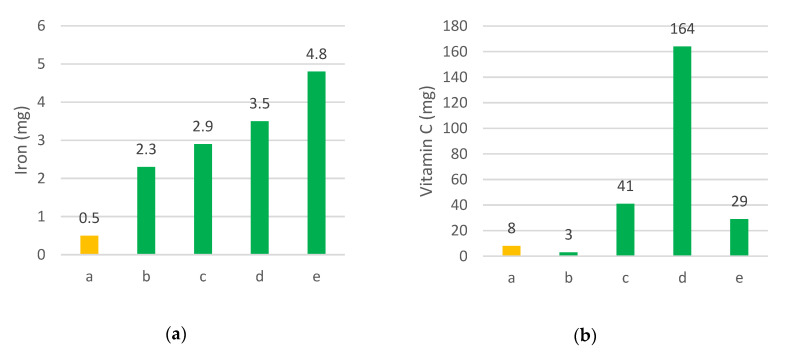
Four wild leafy vegetables from West Sumatra are compared to common lettuce (*Lactuca sativa*) in terms of (**a**) iron content (mg) and (**b**) vitamin C content (mg). In the graphs the letters stand for a lettuce; b vegetable fern “pakis” (*Diplazium esculentum*); c nightshade “leunca/ranti” (*Solanum americanum*), d sweet leaf “katuk/nasi-nasi” (*Sauropus androgynus*), and e water mimosa “komen” (*Neptunia prostrata*). Values are expressed per 100g of fresh, raw ingredient. *Source*: Indonesian Food Composition Data [114].

One of the most recent and comprehensive attempts to fill the evidence gap in food composition data is provided by the GEF-supported Biodiversity for Food and Nutrition Project (BFN). Led by Brazil, Kenya, Sri Lanka, and Turkey, and implemented by Bioversity International with support from the UN Environment Programme (UNEP) and the Food and Agriculture Organization of the United Nations (FAO), the project has generated food composition data for 185 plant species, many of them wild, particularly in Brazil and Turkey [28,150,153]. Because of the high costs associated with food composition analysis, the four countries carried out literature reviews prior to the project to identify information gaps and narrow down the list of potentially interesting species to a practicable sample size for analysis and to select the species with the greatest potential for conservation, domestication/management, promotion and marketing. Following the literature review and identified data gaps, food composition analysis was carried out for those species and nutrients for which information was missing or incomplete. Examples of the high nutrient content of WFPs was demonstrated as part of the BFN project in Brazil and Turkey [28]. Similar results were obtained in Indonesia by reviewing the country’s food composition data [114] in which wild leafy vegetables are reported to contain higher amounts of limiting micronutrients than more commonly consumed greens (Figure 4).

Species selection and prioritization, literature reviews, and generating food composition data is only the first step of a comprehensive and integrated conservation approach.

### 4.3. Collecting, Storing and Maintaining WFP Diversity

Once the species have been identified and prioritized, consideration will need to be given to safeguarding the species for future use, either through ex situ or in situ conservation strategies. Particularly for WFPs, ex situ measures are envisioned as a support to their propagation and reintroduction for habitat restoration [154]. In both cases, to be effective, conservation should involve a wide range of stakeholders working together both in the public and private sectors, across the agricultural and environmental domains [145].

In many cases, governments have established national plant genetic resources programs and seed saving facilities (e.g., gene banks) for ex situ conservation.

However, seed and planting material produced by these “formal” facilities are often inaccessible to smallholder farmers due to strict regulations limiting exchange, little involvement of community actors in the governance, and management of these services [146], as well as imbalances in seed availability, access, and quality for smallholders [155]. However, alternatives do exist. The MGU Useful Plants project, for example, worked closely with communities in Botswana, Kenya, Mali, South Africa, and Mexico to select useful indigenous plants for which high-quality seed collections were established. Seed lots were also banked in the five countries as well as being duplicated and tested at the Millennium Seed Bank in Kew [48,154,156]. Research on seed germination helped support plant propagation activities. The propagules were then planted in community gardens while facilities were established or improved at the local level to facilitate conservation of the prioritized species. Training and knowledge on seed conservation in seed conservation, plant propagation, and planting activities were also provided [49]. This form of conserving WFPs, which takes place in situ, either “on farm” or “in the wild” in natural habitats or protected areas, provides greater opportunity for the involvement of local communities. Once hotspots of WFP occurrence are identified, farmers and indigenous communities living within and around those habitats and protected areas should be involved in conservation activities with due recognition given to their roles and rights in managing WFPs. Further guidance on the establishment of sites for active in situ conservation (i.e., where populations are actively monitored and maintained) of WFPs can be found in the “Voluntary Guidelines on the Conservation and Sustainable Use of Crop Wild Relatives and Wild Food Plants” [145].

Midway between these two conservation approaches are community seed banks or gene banks, which are community-maintained facilities that preserve seeds and other planting materials for local use [146]. These are a collective forms of crop conservation that provide farmers with access to seed, planting material, and traditional knowledge that may otherwise be lost [147]. They also foster community engagement and strengthen the understanding of farmers’ and community’ intellectual property [157]. By documenting and storing biodiversity and associated traditional knowledge, the seed banks also raise awareness of unique biodiversity in a given area. The community-based organizations (CBO) operating in Vihiga County, Western Kenya, have now established their own community seed bank for African indigenous vegetables and legumes [158]. Creating markets for the seeds and planting material can create additional conservation incentives. Such is the case for the communities in Botswana engaged in the MGU UPP who collect the edible seeds of *Tylosema esculentum* (Burch.) A. Schreib (morama bean) for conservation and cultivation, consumption, sale, and processing into numerous marketable food products. Likewise, the Tsetseng community, through their community trust, have become leading innovators in marketable morama products [159].

### 4.4. Domestication Programmes and Guidelines for Sustainable Collection

**Figure 5:**
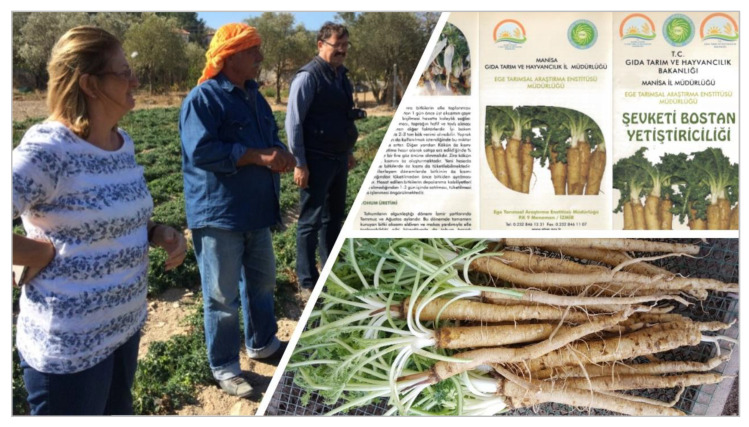
From left to right, top to bottom. BFN Turkey work with farmers to test domesticated golden thistle (*Scolymus hispanicus* L.); the sustainable production guidelines; and harvested golden thistle roots ready for sale Credit: BFN Turkey.

Depending on conservation status and extent of utilization of WFPs, domestication programs may be required to facilitate cultivation of these wild species and thus to ease the pressure on wild populations and rebuild and restore the genetic diversity that has been lost. A successful example is provided by Turkey in its quest to reduce overexploitation of golden thistle (*Scolymus hispanicus* L.). Golden thistle is a flowering plant that is widely consumed across Turkey and is traditionally collected from the wild for its roots and immature leaves that are sold in local markets [62,160]. Selected by the BFN Project as one of the target species for potential commercialization, breeding, and domestication efforts were undertaken by the Aegean Agricultural Research Institute and the University of Anadolu in collaboration with 37 farmers to select, characterize, and evaluate the species [161]. Nurseries established following initial selection of the hardiest plants produced an average yield of 3.9 tons/ha and up to a maximum of 7 tons/ha. A cultivar called “Sari” was registered and seeds distributed to farmers in the İzmir province. Golden thistle is now cultivated on an area of 100 ha^−1^ [161]. To complement seed distribution, guidelines for the sustainable production of golden thistle were also produced to assist farmers in addressing critical aspects such as climate and soil conditions, plant management, harvest, and seed production (Figure 5).

### 4.5. Strengthening Policies in Support of WFP Conservation and Sustainable Use

**Figure 6:**
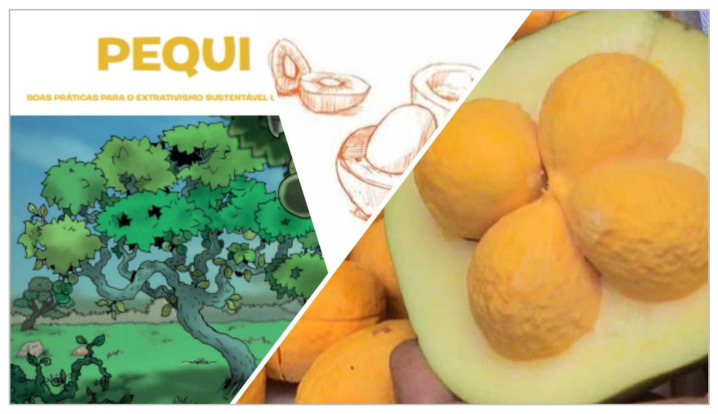
Best practices for the sustainable harvesting and management of pequi (*Caryocar brasiliense* Cambess), common to Brazil’s Cerrado region. The guidelines are produced by the Ministry of Agriculture, Livestock and Supply (MAPA) in support of producers/extractivists intending to take part in the public procurement schemes.

Once baseline data has been gathered, guidelines exist to assist countries in preparing a National Plan for the Conservation and Sustainable Use of Wild Food Plants and crop wild relatives, including setting up a monitoring plan for WFP diversity [145]. The scope of the action plan, its application and effectiveness will very much depend on the national context, the existing policy framework and institutional arrangements, the range of stakeholders involved and their interrelationships, as well as on the resources available. Guidelines are also provided for undertaking preparatory work towards this end [145]. Suggestions are made on how to promote wider use of crop wild relatives and WFPs, but few examples are given to show countries what is practical or actionable. One possibility, which has shown great promise in the BFN Project, is to link producers and collectors to institutional or private sector markets enabling them to benefit from the authorized trade of WFPs. Brazil has used its two largest public procurement programs, the Food Procurement Program (PAA) and the National School Feeding Program (PNAE), to stimulate engagement by family farmers and wild plant collectors (known as extractivists) in sustainable agriculture and the management of Brazilian food diversity, including many WFPs. Both PNAE and PAA are in fact obliged to buy a proportion of the food they distribute from family farmers and pay a 30% bonus for organic or agroecological produce, preferring suppliers from indigenous and traditional communities [162]. PAA also supports activities aimed at the conservation, production, storage, and distribution of local or traditional seed varieties (Beltrame et al., 2020) [28]. Working closely with government actors and using the nutritional data generated as part of the BFN project, BFN Brazil was also able to promote the publication of Ordinance N° 284/2018, which officially recognizes the nutritional and sociocultural value of over 100 plant species native to the Amazon, Caatinga, Atlantic Forest, and Cerrado biomes. This has boosted the market value of native biodiversity including WFPs, with ministries now referring to the list in the “Sociobiodiversity Ordinance” to purchase biodiversity and farmers and collectors eager to join the scheme. In order to do so, however, producers must adhere to procurement regulations, and follow training and guidelines for organic production and the sustainable management of these resources in the wild (Figure 6). Similar linkages were fostered in the other three BFN countries, increasing structured demand for African leafy vegetables in Kenya, for WFPs in Turkey and for native fruits such as wood apple (*Limonia acidissima*) in Sri Lanka including via private sector linkages (see next section).

### 4.6. Raising Public Awareness of the Importance of WFPs

Raising public awareness of the important contribution WFPs can make to diets and livelihoods is another effective way to secure research and policy investments targeting their conservation and use and creating a mutually reinforcing virtuous cycle [163]. This is probably the area in which countries invested in protecting WFPs let lose their creativity and excel in finding ingenious, innovative, and culturally acceptable ways of communicating the importance of WFPs to different target groups. Naturally, collaborating and partnering with the broadest range of stakeholders, e.g., farmer groups, NGOs, private sector enterprises, schools, the media, and ministries will ensure that there is clear and cohesive messaging that is able to reach the widest possible audience.

#### 4.6.1. Youth

**Figure 7:**
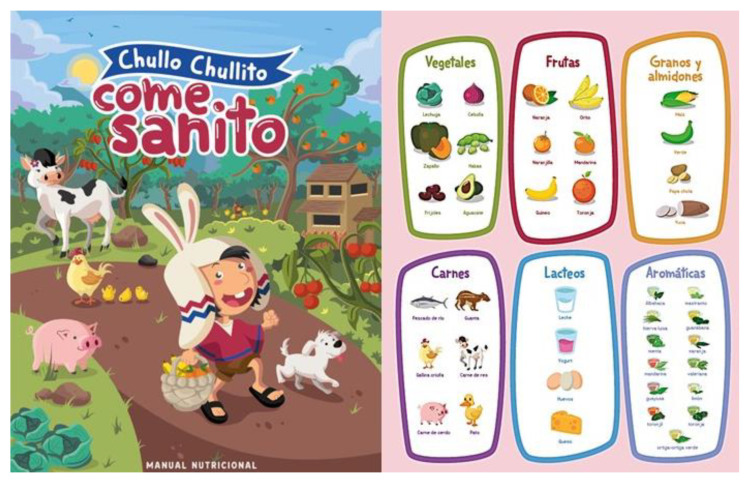
A nutrition education booklet from Ecuador that includes WFPs as a food group. On the left, the cover depicts the forest as an alternative source of foods, mainly fruits, while on the right, five food groups are shown along with a list of 13 WFPs (mostly aromatic plants) used for preparing hot beverages.

As future consumers and protectors of biodiversity, youth are an important target audience for WFP messaging. Awareness raising campaigns can take advantage of recurring activities such as biodiversity festivals or food fairs to organize nature walks or competitions for younger participants [28,150] or join forces with relevant ministries (e.g., Environment, Agriculture, and Education) to introduce messaging around WFP conservation and use in curricular activities and courses (Figure 7). For older students, WFPs offer an interesting opportunity for “greening” vocational training, particularly in the food and beverage sector. In Turkey, to raise the profile of WFPs, the BFN project partnered with the Halim Foçali Vocational School organizing a series of lectures and hands-on activities for 16 student chefs, who were trained to recognize and collect local edible species and use them in their cooking classes. Future plans for the institute include the establishment of an herb garden on the school premises where WFPs will be grown and harvested for use in cooking courses. Interest in the program from the National Education Directorate of Foça has led to plans for extending the training to other schools and officially include traditional WFPs in the school curriculum [150]. School gardens are also an effective way of promoting greater interest in biodiversity and can act as important conduit for improving nutrition, well-being and education of schoolchildren and their families [164], as well as acting as conservation networks for tree genetic resources [165] and reviving traditional food systems and culture [164].

#### 4.6.2. Communities and Households

**Figure 8:**
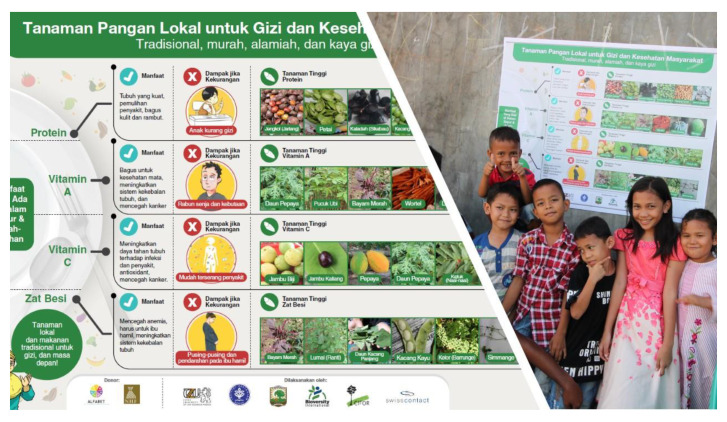
Community poster in local language developed in West Sumatra, Indonesia, as part of the “Food, Agrobiodiversity, and Diet project” explaining the health benefits of local food plants that are rich in protein, vitamin A, vitamin C, and iron, and Mandailing children learning about local foods. Credit: Lukas Pawera.

**Figure 9:**
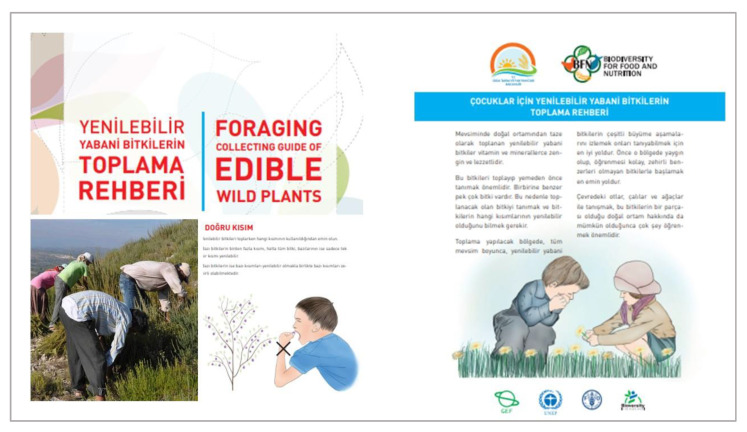
Foragers’ guide to edible wild plants and illustrations taken from “A children’s guide to the collection of wild edibles”, produced by BFN Turkey to complement activities aimed at raising the profile of Turkish WFPs. Credit: BFN Turkey.

As previously mentioned, it will be important to ensure that the main users of this diversity the communities that continue collecting and maintaining WFPs are aware of the species’ nutritional and sociocultural importance. Seasonal food availability booklets and calendars, such as the one shown in Figure 5, and simple, locally appropriate picture posters (Figure 8) can serve the dual purpose of revitalizing the use of WFPs and imparting basic nutrition information derived from national nutrition guidelines. Translated into local languages, these tools can be used by government extension workers and NGO practitioners to offer an overview of local diet quality and consumption patterns derived from baseline assessments and provide recommendations on how WFPs and other local agrobiodiversity can fill existing nutrient gaps. To avoid issues of overharvesting, it will be important, that the above information is complemented by easy-to-understand guidelines on the sustainable collection and management of these species, as shown by the informative brochures that accompanied the revival of WFPs in Turkey (Figure 9).

#### 4.6.3. Policymakers

Policymakers and key change agents who can support the conservation and use of WFPs are to be found within the following sectors: nutrition, health, agriculture, forestry, education, environment, trade, planning, poverty reduction, food security, rural development, economy, and finance at national, regional, and international levels. Whatever their background, for effective decision-making to occur, policymakers need access to timely, independent and reliable information, in a simple and useful form, accompanied by the cost implications of the research, indicating whether it is feasible and affordable [166]. As demonstrated by the endorsement of the “Sociobiodiversity Ordinance” in Brazil, for example, nutrition evidence generated via food composition analysis was critical for expanding the list of sociobiodiversity species to include previously neglected WFPs, and for subsequent policy uptake by national programs dealing with food and nutrition security.

The recognition of WFPs as important elements of healthy diets and rural resilience has thus resulted in increased federal funding (approximately US$6 Million per year) for public procurement programs to purchase sociobiodiversity products directly from family farmers and provided an indication of the untapped market potential of WFPs in institutional markets [167]. The increased appreciation of the role WFPs play in rural diets is also leading to investigations into the affordability of diets that include WFPs [126]. As mentioned earlier, the study carried out in Eastern Baringo, Kenya, has shown that wild plant species, especially vegetables, are able to significantly reduce (by 30–70%) the cost of nutritious meal for vulnerable groups [54]. The tool, which provides an insight into the affordability of nutritious foods, offers a useful entry point for policy discussion around the types of commodities and delivery channels that are likely to achieve nutritional outcomes particularly for the most vulnerable segments of the population [168].

#### 4.6.4. Broader Audiences

**Figure 10:**
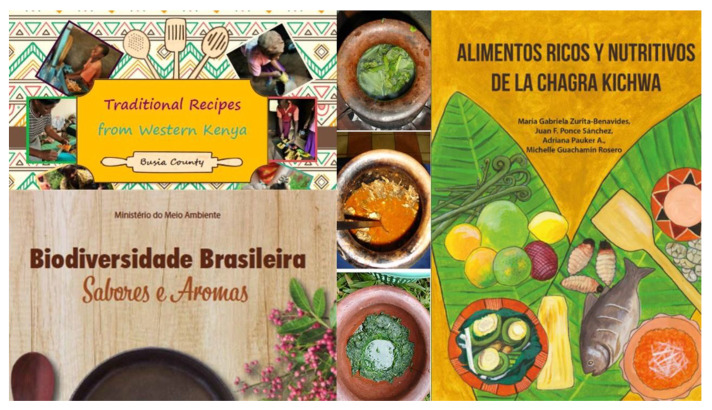
Front covers of recipe books developed as part of the WFP-focused projects in Brazil, Ecuador and Kenya. Credit: BFN Brazil, IKIAM and BFN Kenya.

Recent interest in food and gastronomy programs worldwide has acted as the perfect jumping board for WFPs, particularly in developing countries. Many of the approaches adopted by BFN project countries have extensively been described [28,150,153], and broadly involve communities partnering with celebrity chefs, gastronomists, or taking advantage of existing food festivals to organize information and hands-on events on WFP collection, transformation, and cooking (Figure 10). Innovative approaches for reaching out to broader audiences are described in detail by Gee and Lee (2020) who look at emerging youth-led innovations that can be productively applied to the conservation and sustainable use of food biodiversity, including WFPs. The realms of social media and mobile technology are rapidly evolving, and via mobile apps consumers are now able to (i) find local crops in season and plan grocery purchases, (ii) identify plants through a global photo database, (iii) learn about wild edible plants (Wild Edibles and Foraging Flashcard Lite), (iv) and even trace fresh crops back to farms using blockchain technology. On the production side, a growing number of applications, including in developing countries, offer “smart phone farmers” unprecedented access to crop, field, and market information, which could easily be extended to incorporate WFPs. Gee and Lee [169] also explore the benefits of creating conservation networks for biodiversity through international movements such as via “Campesina” and Slow Food, which can connect different actors who are motivated to improve global and community-based food systems using food biodiversity.

## 5. Conclusions

While WFPs contribute to the diets and livelihoods of millions of people worldwide at the local level, there is still much that we do not yet fully understand about them and thus their role is not fully appreciated. This makes it a challenge when it comes to decisions and actions that might support more effective national and international conservation, sustainable management, and useful strategies for WFPs. Some of these actions are summarized in Table S2. While there are an increasing number of publications outlining the importance of WFPs, usually at a local level, there is largely a scarcity of data and information at a national level, and conservation assessments are still limited. This fails to convey the full contribution that WFPs make to food security and nutrition and the overall importance of these biological resources to national economies in many parts of the world. Furthermore, while we increasingly learn more about some of the threats which impact WFPs, we still know so little about their biology and ecology as well as the dynamics of their use and how climate change is impacting them now and in the future. The integrated conservation approach described in this paper is intended to guide stakeholders in creating plans and strategies to ensure that WFPs are used sustainably and are conserved for generations to come.

In this review we survey the contribution of WFPs to food security, nutrition, and livelihoods in a variety of geographical settings, many of which have benefited from the availability of donor-funded projects and therefore the dedicated attention of researchers and their organizations. It is by no means a comprehensive review. However, the limited cases and examples it highlights clearly demonstrate that the contribution of WFPs to food security, nutrition, and livelihoods is significant. With increased development attention and research investments, including a more effective enabling policy environment, the role of WFPs could be strengthened in the future.

A greater understanding and appreciation, especially by decision-makers, of the nutritional value of WFPs and their contribution to food security and nutrition could see the enhanced inclusion of WFPs in important national nutrition policy instruments such as dietary guidelines, development plans, or in nutrition education and school curricula. Greater use should also go hand in hand with increased research and investments targeting existing biological and ecological knowledge gaps on WFPs, such as plant demographic studies to calculate sustainable harvest levels in the wild or studies on seed biology and ecology to ensure they are adequately conserved ex situ. If WFPs were provided with greater policy recognition and support, especially through policy incentives and the development of innovative market-based demand approaches (with clear benefits arising to custodians), it would help create longer-term economic viability. This, in turn, could help greatly in better linking the conservation of WFPs and their sustainable traditional management and use, something which is currently missing in most national Plant Genetic Resources conservation strategies and action plans.

## Figures and Tables

**Table 1:** Local threat assessments carried out in partnership with local communities have identified increasing dangers to the survival of nutritious and locally important WFPs. A selection from the authors’ project sites is provided along with suggested measures for conservation and sustainable use.

Country	Species Name	Local Name	Edible Use	Main Nutritional Benefits	Habitat	Threat Status (IUCN, Community to Other)	Threats/Suggestion for Conservation	Photo
Brazil	*Astrocaryum aculeatum*	Tucumã	Fruit pulp	Rich in vitamin A as well as lauric, myristic and oleic acid [99]	Amazon rainforest	No IUCN assessment	Habitat loss—deforestation/ Preserve natural habitats	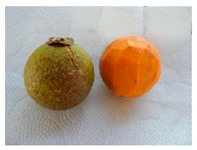 Credit: J. Camillo
	*Euterpe edulis*	Jussara	Fruit pulp consumed as puree, palm heart (discouraged)	The fruit is rich in antioxidants [99]	Dense shady forest (Atlantic forest)	No IUCN assessment, listed as Vulnerable in the Red Book of Brazilian Flora [100]	Habitat loss—deforestation, overharvesting of palm heart/ Preserve natural habitats	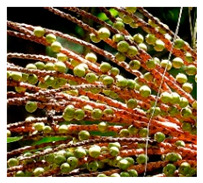 Credit: A. Popovkin
	*Butia eriospatha*	Butiá	Fruit pulp, seed	Good source of fiber, potassium, and vitamin C (equivalent to levels found in oranges) [99]	Highland mixed shady forests (Araucaria forest), around 800–900 m elevation	IUCN—Vulnerable	Habitat loss—deforestation/ Preserve natural habitats	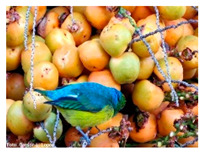 Credit: G. Lopes
	*Dipteryx alata*	Baru nut	Fruit/Nut	High in fiber; the nut is high in quality protein [99]	Tropical savannah (Cerrado)	IUCN—Vulnerable	Habitat loss—deforestation/Preserve natural habitats	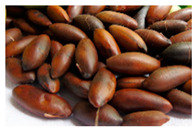 Credit: J. Camillo
	*Hancornia speciosa*	Mangaba	Fruit	Excellent source of vitamin C, folates and a good source of carotenoids and vitamin E [99]	Scrublands (Caatinga) and barren lands in central Brazil	No IUCN assessment	Habitat loss—deforestation/Preserve natural habitats	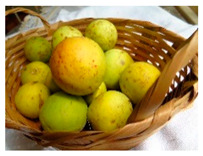 Credit: J. Camillo
Ecuador	*Vasconcellea microcarpa (Carica microcarpa)*	Col de monte	Leaves	N/A	Forest	IUCN—Least concern	Deforestation/Nutrition education needed	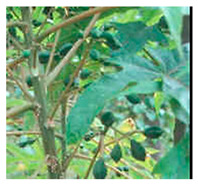 Credit: X. Scheldeman
	*Pouteria multiflora*	Logma	Fruit	N/A	Forest	No IUCN assessment	Deforestation/Nutrition education needed	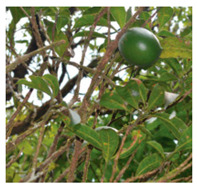 Credit: IKIAM
	*Hypolepis hostilis*	Garabato yuyo	Leafy green vegetable (fern)	N/A	Forest	No IUCN assessment	Loss of traditional food culture /Use as complementary food for infants	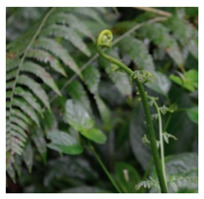 Credit: IKIAM
	*Plukenetia volubilis*	Sachainchi	Nut	Good source of lipids, proteins, and essential amino acids (e.g., cysteine, tyrosine, threonine, and tryptophan), vitamin E and polyphenols [101]	Home garden	No IUCN assessment	Loss of traditional food culture /Use as complementary food for infants	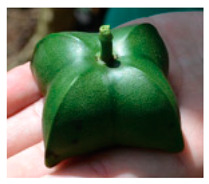 Credit: IKIAM
Fiji/ Samoa	*Caulerpa racemosa*	Nama, Limu	Sea vegetable	Contains proteins, fiber, minerals, vitamins, polyunsaturated fatty acids, and bioactive anti-oxidants [102]	Near reefs, in shallow waters	No IUCN assessment	Unsustainable harvesting, storm surges, cyclones	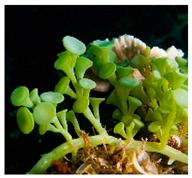 Credit: N.Hobgood
Kenya	*Cleome gynandra*	Spider plant, Ofsaga, saga, liSaga, lisaka	Leaves used as vegetables [103]	High in β-carotene, folic acid, vitamin C, calcium and a good source of vitamin E, iron [104]	Roadsides, field margins, semi-domesticated	No IUCN assessment	No organized collecting missions	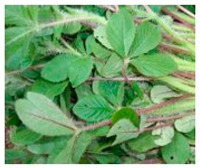 Credit: BFN Kenya
	*Amaranthus tortuosus*	Amaranth, Ekichabo, Dodo	Leaves used as vegetables and seed crushed for flour	Good source of proteins, fibers, calcium, iron, riboflavin, niacin and vitamin C and an excellent source of lysine [104]	Roadsides, field margins, semi-domesticated	No IUCN assessment	No organized collecting missions	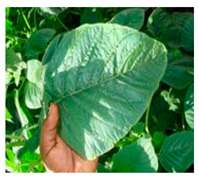 Credit: BFN Kenya
	*Chorchorus olitorius*	Jute mallow, murere	Leaves used as vegetables	High levels of β -carotene, vitamin C, folic acid, calcium and iron [104]	Roadsides, field margins, semi-domesticated	No IUCN assessment	No organized collecting missions	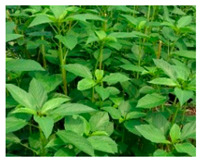 Credit: C. Kerr
Morocco	*Nasturtium officinale*	Watercress, Gernounch	Leafy vegetable	Rich in vitamin K, vitamin A, vitamin C, vitamin B6, manganese, calcium, and folate [105]	Springs, river edges, irrigation canals	IUCN—Least Concern	Paving of irrigation canals may decrease community access, changing diet and preference and leading to decreased use	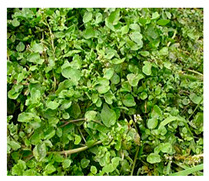 Credit: M. Lavin
	*Malva sylvestris*	Mallow, Tibi, Khobiza, Bakola houra	Leafy vegetable	Strong antioxidant properties, rich in phenols, flavonoids, carotenoids, and tocopherols, alpha-linolenic acid and minerals [106]	Fields, field margins, along irrigation canals and roads	IUCN—Least Concern	Changing diet and preferences may lead to decreased use	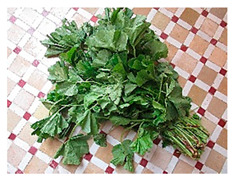 Credit: B. Powell
	*Sideroxylon spinosum*	Argan	Edible oil	Good source of linoleic and oleic fatty acids. Rich source of tocopherol (vitamin E) [107]	Dry forests from the Atlantic coast to 800 m elevation	IUCN criteria at national level—Vulnerable [108]	Social-ecological systems change driven by commodification and globalization	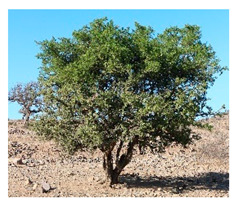 Credit: B. Powell
Niue	*Tacca leontopetaloides*	Kai Niue	Root starch	Good source of carbohydrate, also contains vitamin C, fat, and protein [109]	Uncultivated land	IUCN Least Concern	General lack of information	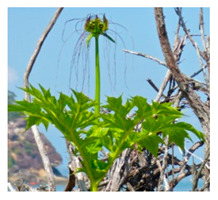 Credit: B. Dupont
Papua New Guinea	*Pandanus brosimos*	Karuka	Fruit (boiled) & extracted nut	Good source of protein and oil especially for highland communities [71]	Forest, high altitudes	No IUCN assessment	No known threat, but general lack of information	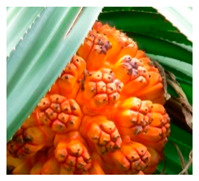 Credit: Green Dean
Turkey	*Scolymus hispanicus*	Golden thistle, Şevketi bostan	Roots and young leaves	Rich in dietary fiber, magnesium and calcium [105,110]	Disturbed habitats and fallow fields	No IUCN assessment	Overharvesting/domestication programs initiated	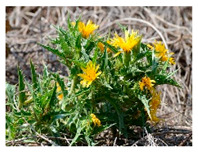 Credit: BFN Turkey
	*Eremurus spectabilis*	Foxtail lily, Çiriş otu	Shoots, buds and young leaves	Rich in antioxidants and minerals [111]. High in vitamin C [112]	Dry and stony grazed hillsides	No IUCN assessment	Overharvesting/domestication programs initiated	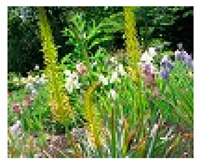 Credit: K.D. Zinnert
West Sumatra, Indonesia	*Elateriospermum tapos*	Tapuih	Seeds consumed raw or fermented	Rich in protein and unsaturated fatty acids [113]	Forest/agroforest	No IUCN assessment	Perceived as rare by local communities/Preserve forest and multi-strata agroforests	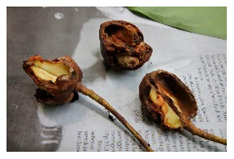 Credit: L. Pawera
	*Mangifera foetida*	Ambacam, Bacang	Fruits consumed raw or cooked	Rich in vitamins A and C [114]	Forest, agroforest, homegardens	IUCN -Least Concern	Perceived as rare by local communities/Preserve forests and multi-strata agroforests	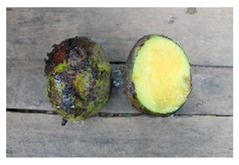 Credit: L. Pawera
	*Diplazium esculentum*	Pakis, Pahu	Young shoots as a vegetable, cooked	Rich in vitamin B9 (folate) [114]	Forests, wetlands	IUCN—Least concern	Relatively common/Preserve forests and wetlands	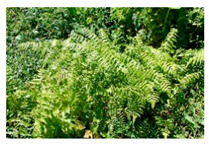 Credit: L. Pawera
	*Ipomoea aquatica*	Kangkung air, Kangkuang liar	Leaves and stems as a vegetable, cooked	Rich in Iron and provitamin A [114]	Rivers, ponds, rice fields	No IUCN assessment	Threatened by overuse of herbicides/Reduce the use of herbicides and keep clean water bodies	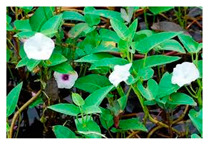 Credit: L. Pawera

**Table 2:** Barriers that hinder the improvement of national policy frameworks towards supporting WFPs.

Awareness	Focus	Financial Support	External Pressures
No adequate data	Mismatch with national priorities	No international financial or donor support	International trade favor R&D on conventional crops
Lack of priority in education and information systems	Limited capacity (institutional, research) to work with WFPs	Weak economies for investing in R&D for WFPs	International R&D priorities influence national priorities

## Supplementary Materials

The following are available online at https://www.mdpi.com/2223-7747/9/10/1299/s1, Table S1: Plant families that include WFPs and semi-cultivated species that are known to contribute to food and nutrition security; Table S2: Summary of actions that can be undertaken across the four pillars by the main stakeholders involved in WFP conservation and use.

## Abbreviations

BFN	Biodiversity for Food and Nutrition Project
CAP	Community action plans
CBD	Convention on Biological Diversity
CBO	Community-based organization
CIAT	International Center for Tropical Agriculture now part of the Alliance of Bioversity International and CIAT
EIARD	European Initiative for Agricultural Research for Development
FAD	Food, Agrobiodiversity and Diet Project
FAO	Food and Agriculture Organization of the United Nations
GEF	Global Environment Facility
GPA	Global Plan of Action for Plant Genetic Resources for Food and Agriculture of the FAO
IKIAM	Universidad Regional Amazónica - Amazon Regional University (Ecuador)
IUCN	International Union for Conservation of Nature
MAPA	Ministério da Agricultura, Pecuária e Abastecimento - Ministry of Agriculture, Livestock and Supply (Brazil)
NBSAPs	National Biodiversity Strategies and Action Plan (of the CBD)
NGO	Non-governmental organization
PAA	Programa de Aquisição de Alimentos - Food Procurement Program (Brazil)
PNAE	Programa nacional de alimentação escolar - National School Feeding Program (Brazil)
PNG	Papua New Guinea
PPF	Plantas Paro o Futuro (Plants for the Future Initiative – Brazil)
RAE	Retinol Activity Equivalents
R&D	Research and Development
SDG	Sustainable Development Goal
SOWBFA	State of the World’s Biodiversity for Food and Agriculture of the FAO
UNEP	UN Environment Programme
UPP	Useful Plants Project, Kew
WFPs	Wild food plants
WLVs	Wild leafy vegetables
