# Supplementary material for: Born to Eat Wild: An Integrated Conservation Approach to Secure Wild Food Plants for Food Security and Nutrition
Source: Plants (Basel). 2020 Oct 1;9(10):1299. doi: 10.3390/plants9101299 (PMC7601573; doi:10.3390/plants9101299)
Supplement: Supplementary file 1 [file plants-09-01299-s001.pdf]

Supplementary Materials

Table S1. Plant families that include wild food plants (WFPs) and semi-cultivated species that are known to contribute to food and nutrition security in project countries and in the study cases mentioned in this review.

Plant Family	Species Name	Common Name	Part Eaten/Commercialized	Range
Amaranthaceae	*Amaranthus tortuosus*	Amaranth	Seeds, leaves	S. America, worldwide
	Chenopodium spp.	Goosefoots	Seeds	Worldwide
Anacardiaceae	*Mangifera foetida*	Ambacam, Bacang *	Fruit	Southeast Asia
Apiaceae	*Foeniculum* sp., cf *F. vulgare*	Fennel	Bulb, leaves	Mediterranean
Apocynaceae	*Hancornia speciosa*	Mangaba *	Fruits	South America—Brazil, Peru, Bolivia, Paraguay
Arecaceae	*Astrocaryum aculeatum*	Tucumã *	Fruits, seeds, leaves, palm heart	S. America—Bolivia, Brazil, Trinidad and Tobago, Venezuela, Guyana, Suriname
	Butia eriospatha	Wooly Jelly Palm	Fruit and seed	S. America—Southern Brazil
	Euterpe edulis	Jussara *	Fruit and palm heart	Brazil
	Euterpe oleracea	Açaí palm	Fruit and seeds	S. America—Brazil
Asphodelaceae	*Eremurus spectabilis*	Foxtail lily	Leaves	Mediterranean
Asteraceae	*Scolymus hispanicus*	Golden thistle	Root, young leaves	Mediterranean
	Porophyllum spp.		Leaves	Mesoamerica
Athyriaceae	*Diplazium esculentum*	Vegetable fern	Young leaves	Asia, Oceania
Brassicaceae	*Nasturtium officinale*	Watercress	Leaves	Europe, Asia
Capparaceae	*Capparis spinosa* and *C. decidua*	Capers	Fruits and flowers	Mediterranean
Caricaceae	*Vasconcellea microcarpa (Carica microcarpa)*	Col de monte *	Fruit, leaves	S. America—Brazil, Bolivia, Peru, Ecuador, Colombia, Venezuela; C. America—Panama
Caryocaraceae	*Caryocar brasiliense*	Pequi *	Fruit, seeds	Brazil
Caulerpaceae	*Caulerpa racemosa*	Sea grapes	Leaves	Worldwide in shallow temperate and tropical seas
Cleomaceae	*Cleome gynandra*	Spider plant	Leaves	Africa
Convolvulaceae	*Ipomoea aquatica*	Water spinach, water morning glory	Young shoots and leaves	Southeast Asia

Dennstaedtiaceae	*Hypolepis hostilis*	Garabato yuyo *	Young shoots	Amazonia
Dioscoreaceae	*Tacca leontopetaloides*	Arrow root	Root	S.E. Asia, Indo-Pacific tropics
Ericaceae	*Arbutus unedo*	Strawberry tree	Fruit	Mediterranean and W. Europe
Euphorbiaceae	*Plukenetia volubilis*	Inca nut	Leaves, seeds	S. America—Brazil, Bolivia, Peru, Ecuador, Colombia, Venezuela, Surinam; Caribbean—Windward Isles
	Elateriospermum tapos	Tapos	Fruits, seeds	S.E. Asia—Thailand, Malaysia, Indonesia
	Schinziophyton rautanenii	Mongongo tree, or manketti tree	Fruit and nut	Sub-Saharan Africa
Fabaceae	*Crotalaria* spp.		Leaves	Mesoamerica
	Dipteryx alata	Baru	Nut	S. America—Paraguay, Bolivia, Peru, Brazil
	Neptunia prostrata (syn. *N. oleracea)*	Water mimosa or sensitive neptunia	Young leaves, shoot tips and young pods	Tropical regions of Africa, S.E. Asia, Australia and S. America
	Tylosema esculentum	Morama tree	Seeds	Southern Africa—Kalahari desert and neighboring sandy regions
Fagaceae	*Quercus* spp.	Oak, acorns	Acorns	Northern hemisphere
Lamiaceae (or Labiatae)	*Mentha* spp.	Mint	Leaves	Mediterranean
	Origanum compactum, O. elongatum	Oregano	Leaves	Mediterranean
	Salvia Rosmarinus (syn. *Rosmarinus officinalis*)	Rosemary	Leaves	Mediterranean
	Salvia spp.	Sage	Leaves	Mediterranean
	Thymus satureioides	Savory thyme	Leaves	Mediterranean
Lecythidaceae	*Bertholletia excelsa*	Brazil nut	Seeds	North and western S. America—Brazil, Venezuela, the Guyanas
Loganiaceae	*Strychnos madagascariensis*	Black monkey orange	Fruit	Eastern and southern Africa—Tanzania, Zambia, Malawi, Zimbabwe, Mozambique, Botswana, Swaziland, S. Africa, Madagascar

Malvaceae	*Adansonia digitata*	Baobab	Fruit, seeds	Tropical Africa—Mauritania to Sudan, south to Angola and Tanzania
	Anoda spp.			Mesoamerica
	Chorchorus olitorius	Jew's mallow	Leaves	Africa, Asia
	Lavandula dentata	Fringed lavender	Leaves	Mediterranean
	Malva sylvestris	Mallow	Leaves	Mediterranean
Moraceae	*Morus* spp.	Mulberries	Fruits	Worldwide in temperate regions
Oleaceae	*Fraxinus dimorpha*	Ash tree	Fruits and seeds	Mediterranean
Pandanaceae	*Pandanus brosimos*	Karuka *	Nuts	Australasia—Papua New Guinea
Phyllanthaceae	*Uapaca kirkiana*	Sugar plum or mahobohobo	Fruit	African tropics
	Sauropus androgynus	Katuk, star gooseberry, or sweet leaf	Leaves, flowers and fruits	E. Asia—India, Bangladesh, S. China to Indonesia, Vietnam
Polygonaceae	*Rumex* spp.	Docks and sorrels	Leaves	Mediterranean
Portulacaceae	*Portulaca* spp.	Purslane	Leaves	Mesoamerica
Rhamnaceae	*Ziziphus jujube*	Jujube	Fruits	S. Asia, S.E. Europe
Rosaceae	*Rubus* spp.	Raspberries, blackberries, and dewberries	Fruits, leaves	Worldwide
Rutaceae	*Limonia acidissima*	Wood-apple and elephant-apple	Fruit	Andaman Islands, Bangladesh, India and Sri Lanka
Sapotaceae	*Sideroxylon spinosum* (syn. *Argania spinosa*)	Argan	Nuts	Northwest Africa—Algeria, Morocco, Western Sahara, Mauritania
	Pouteria multiflora	Bullytree	Fruit	S. America—N. Brazil, Peru, Ecuador, Colombia, Venezuela; C. America—Panama; Caribbean—Trinidad to Jamaica
Solanaceae	*Solanum* spp.	Nightshades	Leaves	Africa, Americas

Note: Species' names were reconciled against Kew's Plants of the World Online (POWO; http://www.plantsoftheworldonline.org/). * Where no common name is available the local name was given.

Table S2. Summary of actions that can be undertaken across the four pillars by the main stakeholders involved in WFP conservation and use.

	Governments	Research Organizations/ Academia	NGOs	WFP collectors	Private sector/Retailers	Consumers
**Inform**	Undertake **policy analysis** of:<br>• International agreements entered into, relevant to WFPs (e.g. CBD, ITPGRFA, GSPC, CITES<br>• National legal frameworks relevant to WFPs<br>• National biodiversity strategy and action plan<br>• National and international information system on WFP	Provide the **background** context for WFPs:<br>• Situation of WFP conservation in the country. Occurrence inside and outside protected areas, in situ actions affecting WFPs; representation in genebanks, assessments of use, trade and threat status<br>• Identification of stakeholders that benefit from use of WFPs (indigenous communities, value chain actors, consumers, breeders)	Identify **key informants** or custodians of biodiversity that can act as **key change agents** or community mobilizers	Engage in citizen science:<br>• provide data on WFP abundance/distribution<br>• participate in food consumption surveys	Companies to review what supply chains rely on wild plant ingredients, assess their ecological and social sustainability	Be mindful of food and well-being products that contain WFP ingredients
	Undertake a **gap analysis** to establish where gaps exist in conservation measures and sustainable use (e.g. governance, land rights, wildlife legislation)	Compile a **national inventory** of WFPs. From the national inventory, select a list of priority species that will be the focus of promotion and conservation activities	**Mobilize** communities to take part in information gathering and **report back** to communities (and governments) once information is analyzed as part of standard development practice			

	Enable effective **regulation** of wild-harvesting and incentivize adherence to **certification** schemes	Review **existing national/regional** data sources on WFPs:<br>• food composition and consumption data<br>• traditional knowledge<br>• importance for food security	Promote the **marketing and consumption** of biodiverse or biodiversity-friendly products	Continue valuing traditional foods and add economic value to WFPs derived products using new information, innovation or processing	Build targets to demonstrate commitment to moving supply chains to verifiable sustainability, and implement third-party standards and certification schemes	Consider the nutritional and health benefits of WFPs and their products
**Value**	Increase **research support** to enhance the visibility of WFPs and their contribution to diets/livelihoods/economies/biodiversity	Carry out a **baseline assessment** for the priority species, including:<br>• ecogeographic status and threat assessment<br>• socio-economic status of consumers, food culture, local knowledge, food security; cultural and health contexts<br>• cultural food list data, food use and nutrient intake patterns	Assist community actors in **value chain development** for WFPs, to create markets for biodiversity-derived products that are collected and managed sustainably	Adhere to certification standards and schemes		
**Conserve**	Put in place **national strategies**, plans or programmes to address the conservation and sustainable use of WFPs	For species requiring *ex situ* conservation, propose **sampling and storage** in national or international genebanks, botanic gardens or other long-term facilities	Advocate policies that favor **sustainable approaches to collection/consumption** (e.g. local food movements; farmers' markets; participatory guarantee systems)	Revive related customary rules or follow new guidelines to sustainably harvest WFPs	Develop/integrate the requirements of sustainable sourcing and trade in existing standards	Reduce pressure on WFPs by consumers choice (e.g. buying fair trade and supporting best practices (such as

						FairWild-certified products)
	Integrate the **management** of WFPs into relevant cross-cutting policies	Propose **complementary actions** to protect WFPs outside protected areas, such as easements, incentive-based schemes or micro-reserves.	Promote **sustainable management and collection** practices based on customary management, national regulations or certification schemes	Engage in community seedbanks and complementary activities to conserve WFPs		
**Educate**	Implement **research and education** programmes with a focus on WFPs	Develop guidelines for **sustainable harvest/management**. For priority species, outline *in situ* conservation action (including threat management), both within protected areas, preferably as a network of genetic reserves, and outside currently protected areas.	Partner with relevant ministries to undertake **nutrition education** programmes that foster the sustainable use of WFPs	Pass on traditional knowledge and train youth to sustainable collect and manage WFPs		Take part in nature walks, food festivals and cooking demonstration that enhance the value of WFPs
